# *Agouti-Signaling Protein* and *Melanocortin-1-Receptor* Mutations Associated with Coat Color Phenotypes in Fallow Deer (*Dama dama*)

**DOI:** 10.3390/genes15081055

**Published:** 2024-08-11

**Authors:** Monika Reissmann, Evelin Ullrich, Uwe Bergfeld, Arne Ludwig

**Affiliations:** 1 Humboldt University Berlin, Thaer-Institute of Agricultural and Horticultural Sciences, 10099 Berlin, Germany; monika.reissmann@hu-berlin.de; 2 Saxon State Office for Environment, Agriculture and Geology, Livestock Husbandry, 04886 Köllitsch, Germany; evelin.ullrich@smekul.sachsen.de (E.U.); uwe.bergfeld@t-online.de (U.B.); 3 Leibniz-Institute for Zoo & Wildlife Research, Department of Evolutionary Genetics, 10315 Berlin, Germany

**Keywords:** coat color, fallow deer, ASIP, MC1R

## Abstract

Four dominant coat color phenotypes are found in fallow deer (*Dama dama*). Brown is the most common. Black, menil, and white occur with varying frequencies. In order to gain insights into the molecular genetic background of these phenotypes, 998 fallow animals (772 brown, 62 black, 126 menil, and 38 white) were examined for mutations in the *ASIP*, *MC1R*, *TYR*, and *SLC45A2* genes. In *ASIP*, two mutations (ASIP-M-E2, located at the boundary from exon 2 to intron 2; and ASIP-M-E3, an InDel of five nucleotides) were found, leading to black fallow deer being either homozygous or heterozygous in combination. There were also two mutations found in *MC1R*. Whereby the mutation MC1R-M1 (leucine to proline, L48P) homozygous leads to a white coat, while the mutation MC1R-M2 (glycine to aspartic acid, G236D) homozygous is associated with the menil phenotype. When both mutations occur together in a heterozygous character state, it results in a menil coat. Since the mutations in the two genes are only present alternatively, 36 genotypes can be identified that form color clusters to which all animals can be assigned. No mutations were found in the *TYR* and *SLC45A2* genes. Our investigations demonstrate that the four dominant coat colors in fallow deer can be explained by *ASIP* and *MC1R* mutations only.

## 1. Introduction

Coat color is an important feature of external appearance. It has a major role in identifying species, can be an expression of sex and strength, and is used for camouflage and protection. Differences in the nucleotide or amino acid sequences of important color genes provide valuable insights into the relationships between different species and their evolution [1,2,3,4]. However, while wild animals usually exhibit a species-typical uniform coloration, domestic animals show an enormous variety of coat colors, which is a key sign of domestication [5,6,7,8]. To date, numerous genes and alleles have been found that determine the type of melanin (eumelanin and pheomelanin, e.g., *ASIP*, and *MC1R*), cause different levels of dilution (e.g., *TYR*, *TYRP1*, *DCT*, *SLC45A2*, *TBX3*, and *SILV*), or regulate the occurrence and function of melanocytes (e.g., *EDNRB*, *KIT*, *KITLG*, *MITF*, and *PAX3*) (reviewed in [9]).

**Figure 1:**
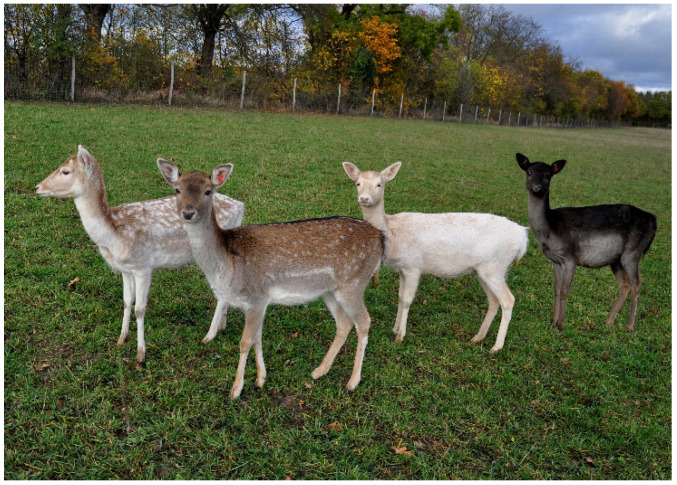
Coat color phenotypes of fallow deer (*D. dama*). From left to right: menil, brown, white and black coat color.

Fallow deer are a valuable game species and are commonly found outside of their historical habitats today [10]. Although the species is reared in farms as fenced animals, fallow deer are rather tamed than domesticated. Notably, the species has a considerable variety of fur colors on farms and in the wild (Figure 1).

The most common brown phenotype is described as a chestnut coat with white mottles, a black dorsal strip, and black strips around the white rump patch. In addition, there are three other main color variants. The darkest of these three phenotypes is a melanistic variant in which the fur is almost completely black except for a slightly lighter belly. Even the normally white rump patch is gray. A light brown variety, whose spots are more distinct than the common variant, is referred to as menil. Finally, there is a very bright, creamy variant that fades to white with age. This variant is not an albino [11] but is sometimes described as leucistic [12]. A mutation in the *MC1R* gene has already been found for this brightest variant [13]. An amino acid exchange from leucine to proline at position 48 is homozygously associated with this extreme coat lightening. Differences can still be seen within the main coat colors. Sometimes, up to 14 variations are distinguished [14]. Although no precise data are available about the frequency of the different color phenotypes, the brown coat color is dominant. However, hunters around the world repeatedly confirm the appearance of black, menil, and white specimens in the wild. The four main colors are described, for example, by the British Deer Society [11] and by the New Zealand Deerstalker Association [12]. Reiner et al. [13] collected samples of white fallow deer in four German hunting areas. In game farms, the occurrence of color phenotypes depends on the preferences of the owners.

The aim of our investigations was to identify additional mutations that explain a wider range of color phenotypes in fallow deer. Considering the knowledge from domestic animals [3], we used a candidate gene approach. The *ASIP* gene stands out as a candidate gene for melanistic coloration. Mutations associated with melanistic variants or diluted phenotypes have already been found in different species (e.g., North American whitetail deer and European red deer [15], horse [16], rabbit [17], sheep [18], European roe deer [19]). The *TYR* and *SLC45A2* genes are involved in pigment dilution, and therefore, reasonable candidates for the menil variant. Numerous mutations have been discovered in the *SLC45A2* gene, particularly in horses [20,21,22,23] but also in tigers [24], which exhibit similar phenotypic brightening. Due to its general importance for color phenotypes, the *MC1R* gene has also been examined for different color variants.

## 2. Materials and Methods

### 2.1. Animals

The investigations were carried out on three groups with a total of 998 animals (Table 1). The first group included 788 fallow deer from the Wein family’s fenced game farm in Nöbeln, Germany, sampled from 2008 to 2020. During this time, there were 17 bucks in reproduction, 14 does introduced before 2008, 670 fawns born between 2008 and 2020, and 87 animals purchased between 2010 and 2020. Color determination was performed for all animals by the breeder. The second group consisted of 17 animals that were shot by hunters in the Mecklenburg Lake District, north-east Germany, in 2022. In this district, hunters have seen and shot not only brown-colored animals, but also black, menil and white fallow deer. The 193 animals in the third group were from the Staar family’s fenced game farm in Gut Hirschaue, Germany. These samples were collected in November and December 2023. Pictures were taken of the furs. Table 1 shows the distribution of coat color phenotypes among the groups. The samples were taken in accordance with the requirements of the German Animal Welfare Act (Landesdirektion Sachsen, Referat Veterinärwesen und Lebensmittelüberwachung, Aktenzeichen N17/14). For DNA extraction, ear punch samples, which accrued during the obligatory insertion of the ear tags in the newborn fawns, as well as skin samples from shot older animals, were used.

### 2.2. DNA Isolation

The DNA was obtained using salt extraction [19], followed by a quality check, and was diluted to 10 ng·µL^−1^ for further analysis. The DNA quality check has been done with the NanoDrop Spektralphotometer (Thermo Fisher Scientific, Darmstadt, Germany) and a 0.8% agarose gel.

### 2.3. Primer Design

The draft genome of European roe deer (*ASIP*: contig DEER_K63_SCAFFOLD265294/GenBank CCMK010265294.1; *MC1R*: contig DEER_K63_SCAFFOLD336989/GenBank CCMK010336989.1; *TYR*: contig DEER_K63_SCAFFOLD47128/GenBank CCMK010047128.1, contig DEER_K63_C29334823/GenBank CCMK013087730.1, contig DEER_K63_SCAFFOLD59893/GenBank CCMK010059893.1, contig DEER_K63_SCAFFOLD129881/GenBank CCMK010129881.1 and *SLC45A2*: contig DEER_K63_SCAFFOLD24609/GenBank CCMK010024609.1, contig DEER_K63_SCAFFOLD135710/GenBank CCMK010135710.1, contig DEER_K63_SCAFFOLD384360/GenBank CCMK010384360.1, DEER_K63_SCAFFOLD54956/GenBank CCMK010054956.1, contig DEER_K63_SCAFFOLD54956/GenBank CCMK010054956.1) was used for primer design with the primer3 software [25,26]. For exon structure, the corresponding ovine sequences from the candidate genes were taken from Ensembl (*ASIP*: ENSOARG00000009483/ENSOART00000010326.1; *MC1R*: ENSOARG00020003524/ENSOART00020005369.2; *TYR*: ENSOARG00000003888/ENSOART00000004229.1; *SLC45A2*: ENSOARG00020018580/ENSOART00020028777.2). Although up to five exons have already been found for the *ASIP* gene, a transcript with three exons was also assumed based on the ovine transcript for the fallow deer. Detailed primer information is provided in the Supplementary Materials (Table S1).

### 2.4. Sequencing

For each color (brown, black, menil, white), candidate genes (2 regions of *MC1R*, 3 regions of *ASIP*, 5 regions of *TYR*, and 6 regions of *SLC45A2*) were sequenced from five animals. In sum, 20 animals were sequenced for the candidate genes. PCR was performed in a total volume of 25 µL, containing 2.5 µL of buffer B, 2.5 mM MgCl_2_, 0.2 mM dNTPs, 0.2 µM of each primer, 1 U FIREPol^®^ DNA Polymerase (Solis Biodyne, Tartu, Estonia), and 50 ng of DNA. The PCR included a denaturation step at 95 °C (5 min), followed by 35 cycles of denaturation at 94 °C (60 s), annealing at the primer-specific temperature (30 s), and elongation at 72 °C (45 s). The final elongation step was carried out at 72 °C for 5 min. In the case of the *ASIP* exon 3, the denaturation step was extended to 15 min, followed by a touch-down PCR from 60 °C to 54 °C at 1 °C per step with an additional 20 cycles at 54 °C. After a PCR check on a 2% agarose gel, the fragments were purified using the HighPrep PCR system (MagBio Genomics Europe GmbH, Kraichtal, Germany) according to the manufacturer’s protocol. Sequencing was carried out with the Sanger method by LGC Genomics (Berlin, Germany).

### 2.5. Genotyping

The three SNP mutations found in the *ASIP* exon 2 (ASIP-M-E2) and the *MC1R* gene (MC1R-M1, MC1R-M2) were genotyped for all animals (*n* = 998) using KBiosciences Allele Specific PCR (KASP) technology. According to the manufacturer’s standard protocol (LCG Genomics, Berlin, Germany), the chemicals and methods were used with StepOnePlus equipment (Thermo Fisher Scientific, Darmstadt Germany) with a 57 °C touch-down PCR for this analysis as described in Kreuzer et al. [27]. The sequenced animals were used as reference samples for the KASP genotyping. To increase the reliability of KASP genotyping, five additional animals for each of the four colors (*n* = 12) were sequenced for each of the three tests for the respective mutation. The primers used can also be found in Supplementary Table S1. An InDel mutation in exon 3 of the *ASIP* gene (ASIP-M-E3) was detected in 126 menil, 38 white, and 62 black animals as well as in 535 brown animals by sequencing with the primer pair A-ASIP-E3. A KASP genotyping test was not possible for this mutation. A total of 237 brown animals were clearly assigned to the DelDel genotype because of their known ancestry, which was determined in a previous project.

## 3. Results and Discussion

### 3.1. Agouti-Signaling Protein (ASIP)

**Figure 2:**
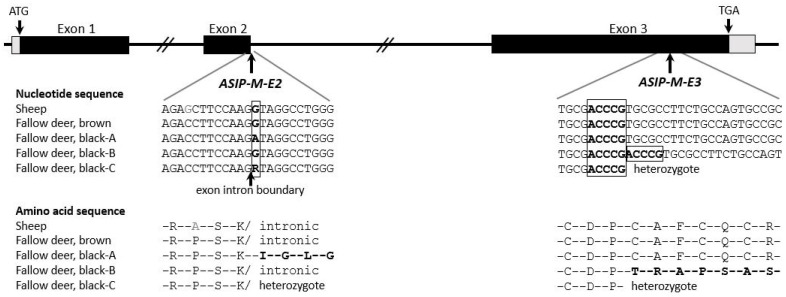
The *ASIP* nucleotide sequences downstream and upstream of the mutations and their effect on the amino acid sequence.

For the *ASIP* gene, the sequence of fallow deer for all three exons and the adjacent intron areas were successfully sequenced (sequences are in Figure S1), leading to the detection of two mutations that are homozygous only in black animals. One mutation, ASIP-M-E2 (g.338G>A), lies exactly at the boundary from exon 2 to intron 2 after amino acid position 75 (Figure 2). This significantly affects the splicing process. If, as a result of failed splicing between exon 2 and exon 3, the intron sequence is read further as exon 2, there would be a stop after 23 amino acids derived from the subsequent intronic sequence. The second mutation, ASIP-M-E3 (g.100-104insACCCG), which was found in exon 3, is an InDel of five nucleotides. This frameshift mutation after amino acid 111 completely changes the following amino acid sequence. In both cases, the cysteine-rich domain on the carboxy-terminal end, which is important for the three-dimensional protein structure over disulfide bonding [15,28], is destroyed. A study in mice confirmed that the third exon of *ASIP* (a fragment of amino acids from position 91 to 131) is important for its antagonistic effect on melanocyte-stimulating hormone [29]. Although all wild fallow deer were brown, the mutation ASIP-M-E2 was found to be heterozygous. The mutation ASIP-M-E3 was not present.

### 3.2. Melanocortin-1-Receptor (MC1R), Tyrosinase (TYR), and Solute Carrier Family 45 Member 2 (SLC45A2)

**Figure 3:**
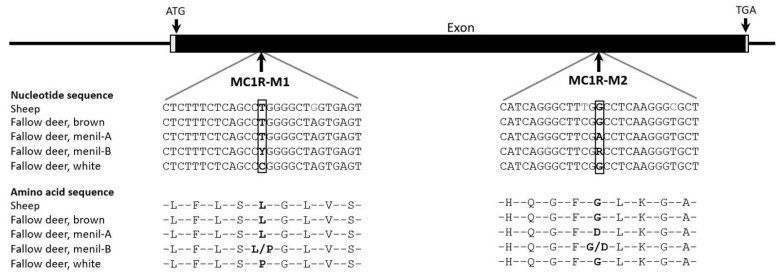
Nucleotide sequences of the fallow deer *MC1R* downstream and upstream of the mutations and their effect on the amino acid sequence.

The complete exon of the *MC1R* gene (954 bp) was also sequenced for the different colored fallow deer (Figure S2); it showed two mutations (Figure 3). The first mutation, MC1R-M1 (g.150T>C), leads to an amino acid change from leucine to proline (L48P) situated in the transmembrane 1 domain [30], while the second mutation, MC1R-M2 (g.714G>A), changes glycine to aspartic acid (G236D) and is located directly on the boundary between intracellular loop 3 and the transmembrane 6 domain [30]. No mutation was found in the *TYR* gene or the *SLC45A2* gene. For this reason, these two genes were not considered further. While the MC1R-M1 mutation was also found to be heterozygous in wild fallow deer, the MC1R-M2 mutation did not occur in these animals. However, it should be noted that only a small number of 17 wild animals were analyzed.

### 3.3. Combined Effect of ASIP and MC1R Mutations on Coat Coloration

Theoretically, the combination of the two *ASIP* mutations, and the two *MC1R* mutations leads to 256 genotypes (Table S2). It is very likely that the two mutations in the *ASIP* gene are located on different homolog chromosomes. There is no evidence that a second mutation has arisen on the chromosome with the existing mutation. If this was the case, the mutations would always have to be inherited in a coupled mode, which is not the case. Since both mutations are very close to each other, an exchange through crossing over is also unlikely. The genotypes of the animals do not indicate such an event in any case. Therefore, they can only occur alternatively. The same applies to the two mutations in the *MC1R* gene. For this reason, there are only 81 possible allelic combinations, of which only 36 are unique if the origin of the allele is not taken into account.

**Figure 4:**
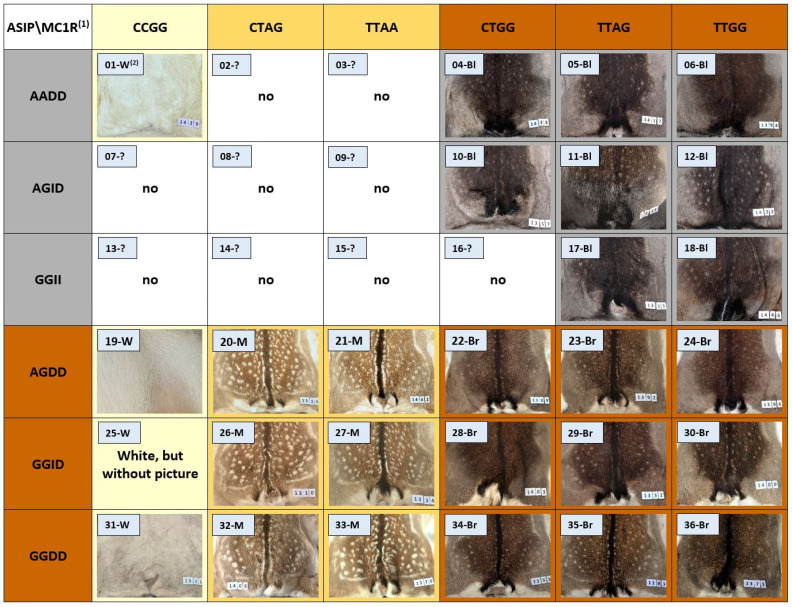
All 36 possible genotypes (numbered consecutively), their coat color phenotype, and a fur picture of a typical animal from this genotype. ^(1)^ *ASIP* alleles in the column (ASIP-M-E2 with A or G, ASIP-M-E3 with I = insertion or D = deletion/non-mutated); *MC1R* alleles in the row (MC1R-M1 with C or T, MC1R-M2 with A or G). ^(2)^ Genotype number and coat color (W = white, Bl = black, Br = brown, M = menil, ? = unknown color).

Figure 4 shows the 36 possible different genotypes as well as the associated phenotypes, whereby only one phenotype can be assigned to each genotype. The genotypes were numbered consecutively to provide an easier overview. The distribution of animals across the different genotypes and phenotypes can be found in Table S3 in the supplement. No representatives could be found for nine genotype variants (02, 03, 07–09, 13–16).

Animals with the same coat color clustered in clear genotype groups. Black fur only occurs if both chromosomes of the *ASIP* gene carry one of the two mutations ASIP-M-E2 g.338A (black-A) or ASIP-M-E3 g.100-104insACCCG homozygote (black-B), as well as g.338A/g.100-104insACCCG heterozygote (black-C), and at least one chromosome in the *MC1R* gene (g.150T and g.714G) is intact (04–06, 10–12, 17–18). All three variants for black color are found in the material. Brown fur, on the other hand, occurs when at least one chromosome in both the *ASIP* and *MC1R* genes does not have a mutation (19, 22–23, 28–30, 34–36). This observation is completely in line with expectations. In many mammals, the Agouti-signaling protein locally blocks the Melanocortin-1 receptor, meaning that only a reddish-brown pigment can be formed. Due to structural changes in the Agouti-signaling protein, this effect is no longer possible, and the formation of black pigment is made possible (proven in an increasing number of species, such as dromedary [31], guinea pig [32], European roe deer [19], Sri Lankan leopard [33], zebu [34]). In peromyscus, two independent mutations in the *ASIP* gene lead to similar melanic coat color [35]. Other studies have shown that reduced expression of Agouti-signaling proteins also leads to increased blackening [36,37].

In many animal species, mutations in the *MC1R* gene lead to a switch from dark brown to black eumelanin to the yellow–reddish pheomelanin pigment synthetic pathway. Examples of this are red cattle [38,39], chestnut horses [40], red foxes [41], and yellow to golden dogs [42]. It has also been described in humans that a loss of function mutation leads to a change in the pheomelanin/eumelanin ratio, which ultimately leads to yellow–reddish hair [43]. When the MC1R-M1 mutation (g.150T>C) in the *MC1R* gene is homozygous for C, white phenotypes likely always arise (01, 19, 25, 31). This MC1R-M1 mutation is the same as that discovered previously [13] via a genome-wide scan and was homozygous for C, which is responsible for the white coat color in fallow deer. Even though this coat color is called white, these animals are not albinos. They have dark eyes, pigments on the nose and on the claws, and the fur of young animals is not completely white. The *MC1R* gene is also not listed as an albino gene [44], but it may have a modifying effect on oculocutaneous albinism type 2 in humans [45]. However, it has been demonstrated several times that the *MC1R* gene plays a central role in pigment production and that mutations can lead to a very strong reduction in entire pigment formation, and thus, to very light and even white fur. In Cashmere goats, black animals exhibit significantly greater expression of the *MC1R* gene than white animals [46]. A missense mutation in *MC1R* (901C/T) in the Arabian camel leads to white animals with dark eyes when the T allele is homozygous or even when the T allele is heterozygous [47]. This would mean that only one mutated *MC1R* allele would lead to a complete failure of pigment production, showing a dominant effect. In this context, it is interesting that the same mutation (901C/T) is found in alpacas, but it only results in lightening in combination with the homozygous occurrence of the G allele in a second mutation (82A/G). It is therefore quite possible that a further mutation is also required in the Arabian camel [48]. It has also been demonstrated in Chinese sheep that mutations in the *MC1R* gene can lead to white hair color. A missense mutation (g.14231948G>A) causes a homozygous G allele in white sheep, while black head sheep are heterozygous [49].

Colored Brazilian creole sheep always show at least one copy of a dominant allele in the *MC1R* gene, while white animals are homozygous for the recessive allele. However, some colored animals also had homozygous recessive alleles. The difference between the two recessive groups was another mutation in the *ASIP* gene [50]. This *ASIP* mutation has also been shown in other studies to be the cause of white coat color in sheep [18,51]. In our samples, the different genotypes of black coat color (07, 13) seemed to have no influence on white coloration.

While the two mutations in the *ASIP* gene lead to an equal darkening of the phenotype, the mutations in *MC1R* have different effects. The A allele of the second mutation, MC1R-M2 (g.714G>A), which was discovered in the *MC1R* gene, does not lead to an almost complete loss of color, but is homozygous for significant lightening (menil-A: 21, 27, 33). When both mutations (MC1R-M1 g.150C and MC1R-M2 g.714A) are heterozygous, there is also a brightening, which is less than when the MC1R-M1 mutation is homozygous but seems to be somewhat stronger than when the MC1R-M2 mutation is homozygous (menil-B: 20, 26, 32). In particular, the dark areas of the dorsal and rump stripe appear lighter than in the menil-A variant. However, this could be the result of clearly reduced pigment production in the presence of the MC1R-M1 mutation. All animals with g.150C/g.150T and g.714A/g.174G as well as g.150T/g.150T and g.714A/g.174A were already classified as menil based on their phenotype prior to genotyping. These animals are characterized by a significant reduction in black pigmentation, especially in the area of the dorsal stripe and the stripes around the rump patch. In the summer, these stripes are caramel [11]. The pictures of our samples in Figure 4 show a slightly darker coat color and even black pigments in the rump patch stripes, but these coats come from animals that were shot in November/December, so they had a winter coat. The brown animals also have a much darker coat than in summer. The difference between menil and brown is clear, however, and is almost more obvious than in the summer coat.

Taken together, four mutations in two genes (*MC1R*; *ASIP*) are responsible for the four basic color phenotypes in fallow deer. Nevertheless, there are further variations within these phenotypes [14,52]. In addition to environmental influences, mutations in other genes are likely responsible for these variations.

## 4. Conclusions

- The four mutations found in this study explain the four main colors of fallow deer. There are certainly other mutations in other genes that lead to further variations.
- White fallow deer are neither albinos (pure white with unpigmented eyes and pink skin) nor leucists (pure white, pink skin, but mostly pigmented eyes and occasionally small pigment spots) since they can produce pigments and possess melanocytes.
- Our results demonstrate that diverse *MC1R* alleles can lead to very different coat colorations, which go far beyond a simple switch from eumelanin to pheomelanin. Previously, it was already concluded that *MC1R* mutations, depending on their position, can result in a non-functional receptor, a constitutively activated receptor, or a hormonally activated receptor [53].

## Figures and Tables

**Table 1:** Number of animals in the different animal groups and distribution by coat color.

Color	Nöbeln				Hunted	Hirschaue	Sum
	Bucks	Old Does	Fawns	Purchase			
Black	1	–	42	8	–	11	62
Brown	13	14	502	75	17	151	772
Menil	3	–	90	4	–	29	126
White	–	–	36	–	–	2	38
Sum	17	14	670	87	17	193	998

## Data Availability

Data are available on request from the authors or found in the Supplementary Materials.

## Supplementary Materials

The following supporting information can be downloaded at: https://www.mdpi.com/article/10.3390/genes15081055/s1, Table S1: Information about sequences, fragment lengths of the PCR products and annealing temperatures of the primers used. Table S2: All 256 possible genotypes from the combination of the four alleles in the *ASIP* and *MC1R* genes. Table S3: Number of animals per genotype and coloration. Figure S1. Sequences of three *ASIP* exons for brown and black fallow deer. Figure S2. Sequences of *MC1R* coding region for brown, menil, and white fallow deer.
