# Supplementary material for: Agouti-Signaling Protein and Melanocortin-1-Receptor Mutations Associated with Coat Color Phenotypes in Fallow Deer (Dama dama)
Source: Genes (Basel). 2024 Aug 11;15(8):1055. doi: 10.3390/genes15081055 (PMC11353312; doi:10.3390/genes15081055)
Supplement: Supplementary file 1 [file genes-15-01055-s001.zip › genes-3149892-supplementary.pdf]

Supplementary Materials

Supplement Table S1. Information about sequences, fragment lengths of the PCR products and annealing temperatures of the primers used.

Gene, part	Name	Sequence 5´ - 3´	Fragment length, bp	Annealing tempera-ture in °C
Sequencing				
ASIP, E1	ASIP-E1 up	GGC ATT ACT GGG GAC CTA TCA AC	940	56
	ASIP-E1 low	CAA CCC TGG CAT GAA AGA ACT A		
ASIP, E2	ASIP-E2 up	GGG ATA CCG GAA ACA CAA GAC CAT	469	56
	ASIP-E2 low	GGC ATG CAA CCC TGG ACA ATC		
ASIP, E3	ASIP-E3 up	CGT CTA GTC CGA GGA GTT CC	298	54
	ASIP-E3 low	GAG CCC CGC CTT TGG AAG		
MC1R, A	MC1R-A up	AAC CCT TCC TGC TCC CTG	600	59
	MC1R-A low	AGA CAC AGC AGG ACG ACC		
MC1R, B	MC1R-B up	GGA TCA TTG CGG CCA TCT G	491	60
	MC1R-B low	GGG CAC ACA ACG GCA CTG		
TYR, E1	TYR-E1 up	TGA CTC GGT AGC TGG AAA GG	962	60
	TYR-E1 low	AGA CCT GCC AGA AGA AGT GA		
TYR, E2	TYR-E2 up	AGA AAG CTG CTT CCT TCA ACA	457	58
	TYR-E2 low	GCA AAC ACT TCT GGG CAC A		
TYR,-E3	TYR-E3 up	TGC TCA CTG GGT ATC TGG AA	397	58
	TYR-E3 low	ACA TCT TCT TGA CAT CCA CTA CA		
TYR, E4	TYR-E4 up	GCA TGG ACT GTG GCT TTA CA	421	58
	TYR-E4 low	AGG TTT TGC ATG CTC TCA GA		
TYR, E5	TYR-E5 up	TGG AGC AAG GAT GAT GGT GA	834	58
	TYR-E5 low	TGT ACA GTC ATG GCA GGA AC		
SLC45A2, E1	SLC45A2-E1 up	ACT CCC AGC TGG CTA ATC TC	672	58
	SLC45A2-E1 low	TCT CCT TGT CCT GAG TTG CA		
SLC45A2, E2	SLC45A2-E2 up	CAG GAT TTA GGA GAC CAA CGG	300	59
	SLC45A2-E2 low	ATC TGA GCG CTG AGA CAC TG		
SLC45A2, E3	SLC45A2-E3 up	TGC TAA TAC AGC TCC TTC TTG C	400	58
	SLC45A2-E3 low	AAG AGT GTT TTC CCT AAG TAA GCA		
SLC45A2, E4-5	SLC45A2-E4-5 up	TCT ACA CTG AAC GCC ACA CC	962	60
	SLC45A2-E4-5 low	CCC AAG AGG AGG AGG CTT AC		
SLC45A2, E6	SLC45A2-E6 up	TGT GAG ATG GTG TGA GGC AT	300	59
	SLC45A2-E6 low	CTC CAG ACG TCA CTC AGC A		
SLC45A2, E7	SLC45A2-E7 up	CAT AGA TCA AAT GCT GTC CCT GT	385	59
	SLC45A2-E7 low	AGT TGA AGT CAT AAC ACA TGG CA		
Genotyping (Mutation)				
ASIP-M-E2	ASIP-M-E2 A1	ACA ATG TGA ACT CCC AGG CCT AT		57 TD(1)
	ASIP-M-E2 A2	CAA TGT GAA CTC CCA GGC CTA C		
	ASIP-M-E2 C	AAG CGG AAA AGA AGA AAA GAC CTT CCA A		
MC1R-M1	MC1R-M1 A1	CTG ACG GGC TCT TTC TCA GCC C		57 TD
	MC1R-M1 A2	TGA CGG GCT CTT TCT CAG CCT		

	MC1R-M1 C	AGC ACA TTC TCC ACG AGA CTC ACT A	
MC1R-M2	MC1R-M2 A1	GGT GGC AGC ACC CTT GAG GT	57 TD
	MC1R-M2 A2	GTG GCA GCA CCC TTG AGG C	
	MC1R-M2 C	AAG AGG CAG CGC CCC ATC CAT	

(1) TD: Touch down PCR

Supplement Table S2. All 256 possible genotypes from the combination of the four alleles in the *ASIP* and *MC1R* genes.

**M/F**[1]	***ADCA***[2, 3]	**ADTA**[4]	***AICA***	***AITA***	***GDCA***	**GDTA**	***GICA***	**GITA**	**ADCG**	**ADTG**	***AICG***	***AITG***	**GDCG**	**GDTG**	**GICG**	**GITG**
ADCA	*AADDCCAA*	*AADDCTAA*	*AAIDCCAA*	*AAIDCTAA*	*AGDDCCAA*	*AGDDCTAA*	*AGIDCCAA*	*AGIDCTAA*	*AADDCCAG*	*AADDCTAG*	*AAIDCCAG*	*AAIDCTAG*	*AGDDCCAG*	*AGDDCTAG*	*AGIDCCAG*	*AGIDCTAG*
**ADTA**	*AADDCTAA*	**AADDTTAA**[5]	*AAIDCTAA*	*AAIDTTAA*	*AGDDCTAA*	**AGDDTTAA**	*AGIDCTAA*	**AGIDTTAA**	**AADDCTAG**	**AADDTTAG**	*AAIDCTAG*	*AAIDTTAG*	**AGDDCTAG**	**AGDDTTAG**	**AGIDCTAG**	**AGIDTTAG**
AICA	*AAIDCCAA*	*AAIDCTAA*	*AAIICCAA*	*AAIICTAA*	*AGIDCCAA*	*AGIDCTAA*	*AGIICCAA*	*AGIICTAA*	*AAIDCCAG*	*AAIDCTAG*	*AAIICCAG*	*AAIICTAG*	*AGIDCCAG*	*AGIDCTAG*	*AGIICCAG*	*AGIICTAG*
AITA	*AAIDCTAA*	*AAIDTTAA*	*AAIICTAA*	*AAIITTAA*	*AGIDCTAA*	*AGIDTTAA*	*AGIICTAA*	*AGIITTAA*	*AAIDCTAG*	*AAIDTTAG*	*AAIICTAG*	*AAIITTAG*	*AGIDCTAG*	*AGIDTTAG*	*AGIICTAG*	*AGIITTAG*
GDCA	*AGDDCCAA*	*AGDDCTAA*	*AGIDCCAA*	*AGIDCTAA*	*GGDDCCAA*	*GGDDCTAA*	*GGIDCCAA*	*GGIDCTAA*	*AGDDCCAG*	*AGDDCTAG*	*AGIDCCAG*	*AGIDCTAG*	*GGDDCCAG*	*GGDDCTAG*	*GGIDCCAG*	*GGIDCTAG*
**GDTA**	*AGDDCTAA*	AGDDTTAA	*AGIDCTAA*	*AGIDTTAA*	*GGDDCTAA*	**GGDDTTAA**	*GGIDCTAA*	**GGIDTTAA**	AGDDCTAG	AGDDTTAG	*AGIDCTAG*	*AGIDTTAG*	**GGDDCTAG**	**GGDDTTAG**	**GGIDCTAG**	**GGIDTTAG**
GICA	*AGIDCCAA*	*AGIDCTAA*	*AGIICCAA*	*AGIICTAA*	*GGIDCCAA*	*GGIDCTAA*	*GGIICCAA*	*GGIICTAA*	*AGIDCCAG*	*AGIDCTAG*	*AGIICCAG*	*AGIICTAG*	*GGIDCCAG*	*GGIDCTAG*	*GGIICCAG*	*GGIICTAG*
**GITA**	*AGIDCTAA*	AGIDTTAA	*AGIICTAA*	*AGIITTAA*	*GGIDCTAA*	GGIDTTAA	*GGIICTAA*	**GGIITTAA**	AGIDCTAG	AGIDTTAG	*AGIICTAG*	*AGIITTAG*	GGIDCTAG	GGIDTTAG	**GGIICTAG**	**GGIITTAG**
**ADCG**	*AADDCCAG*	AADDCTAG	*AAIDCCAG*	*AAIDCTAG*	*AGDDCCAG*	AGDDCTAG	*AGIDCCAG*	AGIDCTAG	**AADDCCGG**	**AADDCTGG**	*AAIDCCGG*	*AAIDCTGG*	**AGDDCCGG**	**AGDDCTGG**	**AGIDCCGG**	**AGIDCTGG**
**ADTG**	*AADDCTAG*	AADDTTAG	*AAIDCTAG*	*AAIDTTAG*	*AGDDCTAG*	AGDDTTAG	*AGIDCTAG*	AGIDTTAG	AADDCTGG	**AADDTTGG**	*AAIDCTGG*	*AAIDTTGG*	AGDDCTGG	**AGDDTTGG**	AGIDCTGG	**AGIDTTGG**
AICG	*AAIDCCAG*	*AAIDCTAG*	*AAIICCAG*	*AAIICTAG*	*AGIDCCAG*	*AGIDCTAG*	*AGIICCAG*	*AGIICTAG*	*AAIDCCGG*	*AAIDCTGG*	*AAIICCGG*	*AAIICTGG*	*AGIDCCGG*	*AGIDCTGG*	*AGIICCGG*	*AGIICTGG*
AITG	*AAIDCTAG*	*AAIDTTAG*	*AAIICTAG*	*AAIITTAG*	*AGIDCTAG*	*AGIDTTAG*	*AGIICTAG*	*AGIITTAG*	*AAIDCTGG*	*AAIDTTGG*	*AAIICTGG*	*AAIITTGG*	*AGIDCTGG*	*AGIDTTGG*	*AGIICTGG*	*AGIITTGG*
**GDCG**	*AGDDCCAG*	AGDDCTAG	*AGIDCCAG*	*AGIDCTAG*	*GGDDCCAG*	GGDDCTAG	*GGIDCCAG*	GGIDCTAG	AGDDCCGG	AGDDCTGG	*AGIDCCGG*	*AGIDCTGG*	**GGDDCCGG**	**GGDDCTGG**	**GGIDCCGG**	**GGIDCTGG**
**GDTG**	*AGDDCTAG*	AGDDTTAG	*AGIDCTAG*	*AGIDTTAG*	*GGDDCTAG*	GGDDTTAG	*GGIDCTAG*	GGIDTTAG	AGDDCTGG	AGDDTTGG	*AGIDCTGG*	*AGIDTTGG*	GGDDCTGG	**GGDDTTGG**	GGIDCTGG	**GGIDTTGG**
**GICG**	*AGIDCCAG*	AGIDCTAG	*AGIICCAG*	*AGIICTAG*	*GGIDCCAG*	GGIDCTAG	*GGIICCAG*	GGIICTAG	AGIDCCGG	AGIDCTGG	*AGIICCGG*	*AGIICTGG*	GGIDCCGG	GGIDCTGG	**GGIICCGG**	**GGIICTGG**
**GITG**	*AGIDCTAG*	AGIDTTAG	*AGIICTAG*	*AGIITTAG*	*GGIDCTAG*	GGIDTTAG	*GGIICTAG*	GGIITTAG	AGIDCTGG	AGIDTTGG	*AGIICTGG*	*AGIITTGG*	GGIDCTGG	GGIDTTGG	GGIICTGG	**GGIITTGG**

(1) Allele combinations of gametes for bucks (M, horizontal) or does (F, perpendicular) for the four mutations

(2) Allele designation: ASIP-M-E2 (A or G), ASIP-M-E3 (I=Insertion, D=Deletion/unmutated), MC1R-M1 (C or T), MC1R-M2 (A or G)

(3) Italic letters in light gray fields (175): Allele combination as well as genotypes impossible because mutations in *ASIP* gene as well as in *MC1R* are not on the same chromosome

(4) Normal letters in uncolored field (45): Duplicated possible genotypes

(5) Bold letters in dark gray fields (36): Unique possible genotypes

Supplement Table S3. Number of animals per genotype and coloration.

Number	Genotype[1]	Phenotype			
		**Black**	**Brown**	**Menil**	**White**
01	AADDCCGG				**1**
02	AADDCTAG				
03	AADDTTAA				
04	AADDCTGG	**5**			
05	AADDTTAG	**13**			
06	AADDTTGG	**23**			
07	AGIDCCGG				
08	AGIDCTAG				
09	AGIDTTAA				
10	AGIDCTGG	**4**			
11	AGIDTTAG	**4**			
12	AGIDTTGG	**9**			
13	GGIICCGG				
14	GGIICTAG				
15	GGIITTAA				
16	GGIICTGG				
17	GGIITTAG	**1**			
18	GGIITTGG	**3**			
19	AGDDCCGG				**7**
20	AGDDCTAG			**19**	
21	AGDDTTAA			**8**	
22	AGDDCTGG		**52**		
23	AGDDTTAG		**64**		
24	AGDDTTGG		**107**		
25	GGIDCCGG				**3**
26	GGIDCTAG			**6**	
27	GGIDTTAA			**5**	
28	GGIDCTGG		**18**		
29	GGIDTTAG		**18**		
30	GGIDTTGG		**32**		
31	GGDDCCGG				**27**
32	GGDDCTAG			**54**	
33	GGDDTTAA			**34**	
34	GGDDCTGG		**118**		
35	GGDDTTAG		**161**		
36	GGDDTTGG		**202**		
**Sum**	**998**	**62**	**772**	**126**	**38**

[1] Allele designation: ASIP-M-E2 (A or G), ASIP-M-E3 (I = insertion, D = deletion/unmutated), MC1R-M1 (C or T), MC1R-M2 (A or G)

Supplement Figure S1. Sequences of three *ASIP* exons for brown and black fallow deer.
yellow underlying: exons
green underlying: mutation ASIP-M-E2 (g.338G>A)
blue underlying: mutation ASIP-M-E3 (g.100-104insACCCG)

Fallow deer, brown: *ASIP* gene exon 1, no mutations in comparison to black animals

AATTCTGCTTAGGCCTTGGGTCTCCTGGAGCCACCGACTTATAAAATGAAACAAAAAGAGCACCCCAC
CCTCTGGAAGGCAGAGACCAATTGCAATGCATTCTTTTTCTCTTTGCAATCTCCCTCCCTTCCTTGTC
TTTCTTCCTCTCCCCTCTCTCCTTTCCTCTCTTTTTCCTTCATGAGTTCTCCTTTCCTGTCCTAAGCC
TTGCTGGTCCTCCAGCTCCACTGGGACACTGGGCTGTGGGCTGGGGGTCAGAGCACCAGCCCAAAGAA
ACATAAAGAAAGCAGGAGGGCACACGCATTTGCCAGAACCCCCGGCCCACCTGACTGCCTTCTCTGTC
CCTCTCAGGCCTCCTGGG**ATG**GACGTCAGCCGCCTCCTCCTGGCTACCTTGCTGGCCTGCCTGTGCTT
CCTCACTGCCTACAGCCACCTGGCACCTGAGGAAAAGCCCAGAGATGAAAGGAACCTGAAGAACAACT
CTTCCATGAACCTGTTGGATTTCCCTTCTGTCTCTATTGTGGGTAAGTAGTCTTACCCACTGGCTGGC
CTGGGGCCCAGCCTCTGGGCTCTGGCCCATGAGAAGGGGCTAAAGGGGGTCAAACACTCCCCAAGCCG
CTATCAGGATCCATTGCCGTAGGCCAGAGCTCCTTTGTGCTCATTCCTCAGAACAATTCTGGAGGAAT
CAAGTGCCCCTTAACTCATTTGGTTAAGAAAGCTCTGCCTCTCACCAATCTAATATGCAGCTTAGCAA
GTTAGAGGCTCTGAGAAACCCTAAAGTCAAGACACCTGGTGAATTTGGTTTAACTTGGCATTTCTGAA
ACTTACTGAATCATATAACTCCTATCAATGGTAAAATTTTTAGAAAATTCTGCAGAATATAACCAAGT
CTGGGATGAAC

Fallow deer, brown: *ASIP* gene exon 2 (ASIP-M-E2 homozygous G)

CCTTGTATGAGACACTGAGTCCATTTTCCAGGGCCTTCTTGGATTTTCCCTTTAGCTTTCCTTCAGAG
GACTCTTTGCTCTCAGTCTCTGGCTCCAGGTCTGCAGCCAGGCTGCAGAAGCTGCTGGCCTAAGTCCC
AAGATGTGATCTATCCAACCAAACCTTCATCCTCTCCCCAACCCTGGGGCTTCCTAGAGCCCTTTCTG
CTCCTCCCACTTCACTGCAGTTAAGGATCCTGAGAAAATGGCTTTGTTCCTTCTGTCTCTCTCTGAAG
CACTGAACAAGAAATCCAAAAAGATCAGCAGAAATGAAGCGGAAAAGAAGAAAAGACCTTCCAAG**G**TA
GGCCTGGGAGTTCACATTGTCAGGATGGGACTGGACTTAAAGGGGGGGGACACCCAAACTCTCGCTA
GGAACTAAATGAAA

Fallow deer, black-A: *ASIP* gene exon 2 (ASIP-M-E2 homozygous A)

CCTTGTATGAGACACTGAGTCCATTTTCCAGGGCCTTCTTGGATTTTCCCTTTAGCTTTCCTTCAGAG
GACTCTTTGCTCTCAGTCTCTGGCTCCAGGTCTGCAGCCAGGCTGCAGAAGCTGCTGGCCTAAGTCCC
AAGATGTGATCTATCCAACCAAACCTTCATCCTCTCCCCAACCCTGGGGCTTCCTAGAGCCCTTTCTG
CTCCTCCCACTTCACTGCAGTTAAGGATCCTGAGAAAATGGCTTTGTTCCTTCTGTCTCTCTCTGAAG
CACTGAACAAGAAATCCAAAAAGATCAGCAGAAATGAAGCGGAAAAGAAGAAAAGACCTTCCAAG**A**TA
GGCCTGGGAGTTCACATTGTCAGGATGGGACTGGACTTAAAGGGGGGGGACACCCAAACTCTCGCTA
GGAACTAAATGAAA

Fallow deer, brown: *ASIP* gene exon 3 (ASIP-M-E3 homozygous without insertion)

CAGGCAGGGGTGAGGACAACTGGGGCGGACGTGGATGGCTCGGGCAGCCCCGGCATTTCCCCGCAGAG
AAAGGCTCCGATGAAGAACGTGGCACGGCCCCGGCCCCCGCCGCCTACCCCCTGCGTGGCCACCCGCG
ACAGCTGCAAGCCTCCCGCGCCCGCGTGCTGCG**ACCCG**TGCGCCTTCTGCCAGTGCCGCTTCTTCCGC
AGCGTCTGCTCCTGCCGCGTGCTCAACCCCACCTGC**TGA**GCGCGCCTCAGGGCGGG

Fallow deer, black-B: *ASIP* gene exon 3 (ASIP-M-E3 homozygous with insertion)

CAGGCAGGGGTGAGGACAACTGGGGCGGACGTGGATGGCTCGGGCAGCCCCGGCATTTCCCCGCAGAG
AAAGGCTCCGATGAAGAACGTGGCACGGCCCCGGCCCCCGCCGCCTACCCCCTGCGTGGCCACCCGCG
ACAGCTGCAAGCCTCCAGCGCCCGCGTGCTGCG**ACCCGACCCG**TGCGCCTTCTGCCAGTGCCGCTTCT
TCCGCAGCGTCTGCTCCTGCCGCGTGCTCAACCCCACCTGC**TGA**GCGCGCCTCAGGGCGGG

Fallow deer, black-C: *ASIP* gene exon 2 and exon 3 (ASIP-M-E2 heterozygous A and G, ASIP-M-E3 heterozygous with and without insertion)

Exon 2
CCTTGTATGAGACACTGAGTCCATTTTCCAGGGCCTTCTTGGATTTTCCCTTTAGCTTTCCTTCAGAG
GACTCTTTGCTCTCAGTCTCTGGCTCCAGGTCTGCAGCCAGGCTGCAGAAGCTGCTGGCCTAAGTCCC
AAGATGTGATCTATCCAACCAAACCTTCATCCTCTCCCCAACCCTGGGGCTTCCTAGAGCCCTTTCTG
CTCCTCCCACTTCACTGCAGTTAAGGATCCTGAGAAAATGGCTTTGTTCCTTCTGTCTCTCTCTGAAG
CACTGAACAAGAAATCCAAAAAGATCAGCAGAAATGAAGCGGAAAAGAAGAAAAGACCTTCCAAG**R**TA
GGCCTGGGAGTTCACATTGTCAGGATGGGACTGGACTTAAAGGGGGGGGACACCCAAACTCTCGCTA
GGAACTAAATGAAA

Exon 3
CAGGCAGGGGTGAGGACAACTGGGGCGGACGTGGATGGCTCGGGCAGCCCCGGCATTTCCCCGCAGAG
AAAGGCTCCGATGAAGAACGTGGCACGGCCCCGGCCCCCGCCGCCTACCCCCTGCGTGGCCACCCGCG
ACAGCTGCAAGCCTCCAGCGCCCGCGTGCTGCG**ACCG/ACCCGACCCG**TGCGCCTTCTGCCAGTGCCG
CTTCTTCCGCAGCGTCTGCTCCTGCCGCGTGCTCAACCCCACCTGC**TGA**GCGCGCCTCAGGGCGGG

Supplement Figure S2. Sequences of *MC1R* coding region for brown, menil, and white fallow deer.
yellow underlying: ATG and Stop
green underlying: mutation MC1R-M1 (g.150T>C)
blue underlying: mutation MC1R-M2 (g.714G>A)

Fallow deer, brown: MC1R gene (MC1R-M1 homozygous T, MC1R-M2 homozygous G)

CGGGATG**ATG**CCTGTGCTTGGCTCCCAGAGGCGGCTGCTGGGTTCCCTTAACTGCACGCCTCCAGCCA
CCTTCCCCCTCACGCTGGCCCCCAACCGGACGGGGCCCCAGTGCCTGGAGGTGTCCATCCCTGACGGG
CTCTTTCTCAGCC**T**GGGGCTAGTGAGTCTCGTGGAGAATGTGCTGGTGGTGGCCGCCATCGCCAAGAA
CCGCAACCTGCACTCCCCCATGTACTACTTCATCTGCTGCCTGGCCATGTCCGACCTGCTGGTAAGTG
TCAGCAATGTGCTGGAGACAGCGGTCATGCTGCTGCTGGAGGCTGGTGCCCTGGCCGCCCAGGCAGCT
GTGGTGCAACAGCTGGACAATGTCATCGACGTGCTTATCTGTGGCTCCATGGTGTCCAGCCTCTGCTT
CCTGGGCGCCATCGCTGTGGACCGCTACATCTCCATCTTCTACGCCCTGCGGTACCACAGTGTCGTGA
CACTGCCCCGGGCGTGGCGGATCATTGCGGCCATCTGGGTGGCCAGCATCCTCACCAGCCTGCTCTTC
ATCACCTACTACAACCACACGGTCGTCCTGCTGTGTCTTGTTGGCTTCTTCATAGCCATGCTGGCCCT
CATGGCCGTCCTCTACGTCCATATGCTGGCCCGGGCGTGCCAGCATGCCCGGGGCATTGCCCGGCTCC
AGAAGAGGCAGCGCCCCATCCATCAGGGCTTCG**G**CCTCAAGGGTGCTGCCACCCTCACCATCCTGCTG
GGCGTCTTTTTCCTCTGCTGGGGCCCCTTCTTCCTGCACCTCTCGCTCATCGTCCTCTGCCCCCAGCA
TCCCACCTGTGGCTGCATCTTCAAGAACTTCAACCTCTTTCTGGCCCTCATCATTTGCAACGCCATCG
TGGACCCCCTCATCTACGCCTTCCGCAGCCAGGAGCTCCGGAAGACGCTCCAAGAGGTGCTGCAGTGC
TCCTGG**TGA**GGCTGG

Fallow deer, menil-A: MC1R gene (MC1R-M1 homozygous T, MC1R-M2 homozygous A)

CGGGATG**ATG**CCTGTGCTTGGCTCCCAGAGGCGGCTGCTGGGTTCCCTTAACTGCACGCCTCCAGCCA
CCTTCCCCCTCACGCTGGCCCCCAACCGGACGGGGCCCCAGTGCCTGGAGGTGTCCATCCCTGACGGG
CTCTTTCTCAGCC**T**GGGGCTAGTGAGTCTCGTGGAGAATGTGCTGGTGGTGGCCGCCATCGCCAAGAA
CCGCAACCTGCACTCCCCCATGTACTACTTCATCTGCTGCCTGGCCATGTCCGACCTGCTGGTAAGTG
TCAGCAATGTGCTGGAGACAGCGGTCATGCTGCTGCTGGAGGCTGGTGCCCTGGCCGCCCAGGCAGCT
GTGGTGCAACAGCTGGACAATGTCATCGACGTGCTTATCTGTGGCTCCATGGTGTCCAGCCTCTGCTT
CCTGGGCGCCATCGCTGTGGACCGCTACATCTCCATCTTCTACGCCCTGCGGTACCACAGTGTCGTGA
CACTGCCCCGGGCGTGGCGGATCATTGCGGCCATCTGGGTGGCCAGCATCCTCACCAGCCTGCTCTTC
ATCACCTACTACAACCACACGGTCGTCCTGCTGTGTCTTGTTGGCTTCTTCATAGCCATGCTGGCCCT
CATGGCCGTCCTCTACrTCCATATGCTGGCCCGGGCGTGCCAGCATGCCCGGGGCATTGCCCGGCTCC
AGAAGAGGCAGCGCCCCATCCATCAGGGCTTCG**A**CCTCAAGGGTGCTGCCACCCTCACCATCCTGCTG
GGCGTCTTTTTCCTCTGCTGGGGCCCCTTCTTCCTGCACCTCTCGCTCATCGTCCTCTGCCCCCAGCA
TCCCACCTGTGGCTGCATCTTCAAGAACTTCAACCTCTTTCTGGCCCTCATCATTTGCAACGCCATCG
TGGACCCCCTCATCTACGCCTTCCGCAGCCAGGAGCTCCGGAAGACGCTCCAAGAGGTGCTGCAGTGC
TCCTGG**TGA**GGCTGG

Fallow deer, menil-B: MC1R gene (MC1R-M1 heterozygous C and T, MC1R-M2 heterozygous A and G)

CGGGATG**ATG**CCTGTGCTTGGCTCCCAGAGGCGGCTGCTGGGTTCCCTTAACTGCACGCCTCCAGCCA
CCTTCCCCCTCACGCTGGCCCCCAACCGGACGGGGCCCCAGTGCCTGGAGGTGTCCATCCCTGACGGG
CTCTTTCTCAGCC**Y**GGGGCTAGTGAGTCTCGTGGAGAATGTGCTGGTGGTGGCCGCCATCGCCAAGAA
CCGCAACCTGCACTCCCCCATGTACTACTTCATCTGCTGCCTGGCCATGTCCGACCTGCTGGTAAGTG
TCAGCAATGTGCTGGAGACAGCGGTCATGCTGCTGCTGGAGGCTGGTGCCCTGGCCGCCCAGGCAGCT
GTGGTGCAACAGCTGGACAATGTCATCGACGTGCTTATCTGTGGCTCCATGGTGTCCAGCCTCTGCTT
CCTGGGCGCCATCGCTGTGGACCGCTACATCTCCATCTTCTACGCCCTGCGGTACCACAGTGTCGTGA
CACTGCCCCGGGCGTGGCGGATCATTGCGGCCATCTGGGTGGCCAGCATCCTCACCAGCCTGCTCTTC
ATCACCTACTACAACCACACGGTCGTCCTGCTGTGTCTTGTTGGCTTCTTCATAGCCATGCTGGCCCT
CATGGCCGTCCTCTACrTCCATATGCTGGCCCGGGCGTGCCAGCATGCCCGGGGCATTGCCCGGCTCC
AGAAGAGGCAGCGCCCCATCCATCAGGGCTTCG**R**CCTCAAGGGTGCTGCCACCCTCACCATCCTGCTG
GGCGTCTTTTTCCTCTGCTGGGGCCCCTTCTTCCTGCACCTCTCGCTCATCGTCCTCTGCCCCCAGCA
TCCCACCTGTGGCTGCATCTTCAAGAACTTCAACCTCTTTCTGGCCCTCATCATTTGCAACGCCATCG
TGGACCCCCTCATCTACGCCTTCCGCAGCCAGGAGCTCCGGAAGACGCTCCAAGAGGTGCTGCAGTGC
TCCTGG**TGA**GGCTGG

Fallow deer, white: MC1R gene (MC1R-M1 homozygous C, MC1R-M2 homozygous G)

CGGGATG**ATG**CCTGTGCTTGGCTCCCAGAGGCGGCTGCTGGGTTCCCTTAACTGCACGCCTCCAGCCA
CCTTCCCCCTCACGCTGGCCCCCAACCGGACGGGGCCCCAGTGCCTGGAGGTGTCCATCCCTGACGGG
CTCTTTCTCAGCC**C**GGGGCTAGTGAGTCTCGTGGAGAATGTGCTGGTGGTGGCCGCCATCGCCAAGAA
CCGCAACCTGCACTCCCCCATGTACTACTTCATCTGCTGCCTGGCCATGTCCGACCTGCTGGTAAGTG
TCAGCAATGTGCTGGAGACAGCGGTCATGCTGCTGCTGGAGGCTGGTGCCCTGGCCGCCCAGGCAGCT
GTGGTGCAACAGCTGGACAATGTCATCGACGTGCTTATCTGTGGCTCCATGGTGTCCAGCCTCTGCTT
CCTGGGCGCCATCGCTGTGGACCGCTACATCTCCATCTTCTACGCCCTGCGGTACCACAGTGTCGTGA
CACTGCCCCGGGCGTGGCGGATCATTGCGGCCATCTGGGTGGCCAGCATCCTCACCAGCCTGCTCTTC
ATCACCTACTACAACCACACGGTCGTCCTGCTGTGTCTTGTTGGCTTCTTCATAGCCATGCTGGCCCT
CATGGCCGTCCTCTACGTCCATATGCTGGCCCGGGCGTGCCAGCATGCCCGGGGCATTGCCCGGCTCC
AGAAGAGGCAGCGCCCATCCATCAGGGCTTCG**G**CCTCAAGGGTGCTGCCACCCTCACCATCCTGCTG
GGCGTCTTTTTCCTCTGCTGGGGCCCCTTCTTCCTGCACCTCTCGCTCATCGTCCTCTGCCCCCAGCA
TCCCACCTGTGGCTGCATCTTCAAGAACTTCAACCTCTTTCTGGCCCTCATCATTTGCAACGCCATCG
TGGACCCCCTCATCTACGCCTTCCGCAGCCAGGAGCTCCGGAAGACGCTCCAAGAGGTGCTGCAGTGC
TCCTGG**TGA**GGCTGG
